# A rare case of mucoepidermoid carcinoma ex-pleomorphic adenoma of the hard palate

**DOI:** 10.4317/jced.59982

**Published:** 2023-05-01

**Authors:** Daniel-Pitanga-de Sousa Nogueira, José-Lacerda Chagas-Neto, Décio-Fragata Silva, Ivisson-Xavier Duarte, John-Lennon-Silva Cunha, Agenor-Gomes dos Santos-Neto, Rogério-Oliveira Gondak, Ricardo-Luiz-Cavalcanti de Albuquerque-Júnior

**Affiliations:** 1 School of Dentistry, Tiradentes University, Aracaju, Sergipe, Brazil; 2 School of Dentistry, State University of Paraíba, Campina Grande, Paraíba, Brazil; 3 Institute of Research and Technology, Laboratory of Morphology and Experimental Pathology, Aracaju, Sergipe, Brazil; 4 Department of Pathology, Federal University of Santa Catarina

## Abstract

Carcinoma Ex-Pleomorphic Adenoma (CExPA) is a salivary gland carcinoma derived from a primary or recurrent benign pleomorphic adenoma (PA) extremely rare in minor salivary glands. In this paper, we report the case of a male afrodescendant patient, 37 years old, presenting a palatal irregular nodular lesion with approximately 3.5 cm diameter. The lesion had over two years of evolution, but started growing faster and presenting pain and ulceration in the last two months. The incisional biopsy revealed a typical pleomorphic adenoma with focal areas of nests of epidermoid and mucous cells, as well as microcyst formations, resembling the mucoepidermoid carcinoma (MEC). Immunohistochemical analysis revealed positivity for CK7, CK13, CK 14, p63 and Ki67 (about 30%), whereas α-SMA was restricted to the PA component. The diagnosis was CExPA (MEC-type). A discussion on the histopathological and immunohistochemical criteria for differential diagnosis of CExPA is provided in this work, hoping to contribute to a better knowledge and understanding of this rare malignant tumor.

** Key words:**Salivary gland neoplasms, pleomorphic adenoma, adenocarcinoma, mucoepidermoid carcinoma, pathology, differential diagnosis.

## Introduction

Carcinoma ex-pleomorphic adenoma (CExPA) is defined as a malignant epithelial tumor arising from a primary or recurrent benign pleomorphic adenoma (1). CExPA is still considered a challenging diagnosis in oral pathology due to the overlap of morphological findings with other salivary gland neoplasms (2), and it can present histological features of salivary duct carcinoma, adenocarcinoma not otherwise specified, myoepithelial carcinoma and, mainly, adenoid cystic carcinoma and mucoepidermoid carcinoma, in addition to extensive areas of typical pleomorphic adenoma (3). Although CExPAs comprise about 3% of all salivary gland neoplasms, it is extremely rare in oral sites (4).

## Case Report

**Figure 1:**
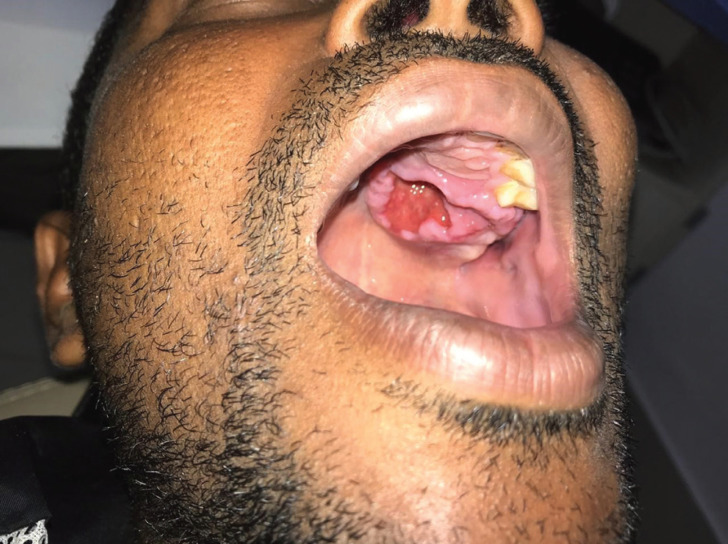
Intraoral examination showed a nodular lesion on the right side of palate, with approximately 3,5 cm diameter, ill-defined limits and ulcerated surface.

**Figure 2:**
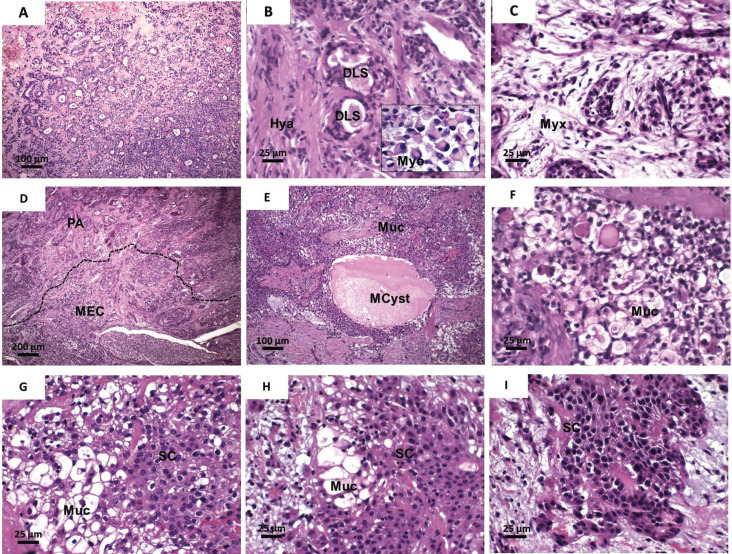
HE-stained histological sections of the tumor. (A) Areas of typical pleomorphic adenoma comprised of proliferating duct-like structures and myoepithelial cells (100 x). (B) Double-cell layer lining of the duct-like structures (DLS) in a hyalinized stromal (Hya) (400 x). In detail, plasmacytoid myoepithelial cells (800 x). (C) Areas of stromal myxoid change (Myx) (400 x); (D) Areas of transition (dotted line) from pleomorphic adenoma (PA) to mucoepidermoid carcinoma (MEC) showing abundance of mucous cells (40 x). (E) MEC-areas composed of sheets of mucous cells (Muc) and occasional microcyst formation (MCyst) (100 x). (F) Detail of mucous cells with goblet appearance (400 x). (G) and (H) Phenotype transition from mucous (Muc) to squamous cells (SC) (400 x). (I) Nests of atypical squamous cells (400 x).

**Figure 3:**
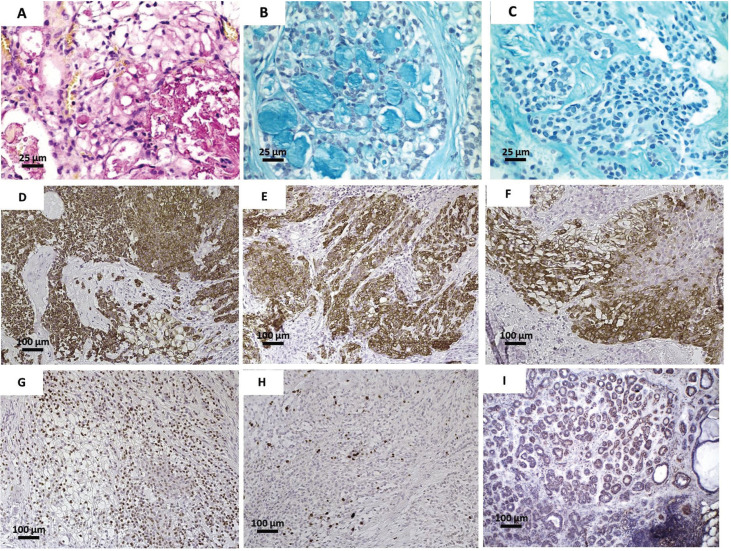
Histological sections showing mucoid material in the cytoplasm of mucous cells and with duct-like structures positively stained for (A) mucicarmine and (B) alcian blue (400 x). (C)Squamous cells negative for alcian blue stain, indicating lack of mucoid material. (D) Tumor cells showing intense immunohistochemical positivity for (D) CK7, (E) CK13, (F) CK14, (G) p63 and (H) Ki67 (100 x). (I) Positivity for α-SMA limited to duct-like structures in the pleomorphic adenoma component (100 x).

A 37 year-old afrodescendant male presented a chief complaint of pain in the palate. The intraoral clinical examination showed a nodular lesion on the right side of palate, with approximately 3.5 cm diameter and ill-defined limits and ulcerated surface (Fig. 1). The lesion had over two years of evolution. However, in the last two months, the patient reported that the lesion started to grow faster, in addition to presenting pain and ulceration. An incisional biopsy was performed, whose histopathological analysis revealed areas of typical pleomorphic adenoma, represented by islands and trabeculae of epithelial cells, some of them with plasmacytoid myoepithelial differentiation, in addition to numerous duct-like structures lined by a double cell layer. The stromal connective tissue was fibrous with hyaline and myxoid changes (Fig. 2A-C). In focal areas, nests of epithelial cells with epidermoid and mucous differentiation, as well as small optically clear cells and microcysts lined by intermediate and mucous cells, were observed. Epidermoid cells showed weak to moderate atypia (Fig. 2D-I). Mucoid material was positive for mucicarmine and Alcian blue stain (Fig. 3A-C). Immunohistochemical analysis (Fig. 3D I) showed strong positivity for CK 7, CK13, CK14 and p63, whereas ki-67 was positive in approximately 30% of tumor cells. Immunoexpression of α-SMA was seen in PA areas but not in MEC areas. The final diagnosis was carcinoma ex-pleomorphic adenoma (CExPA MEC-type). Tumor resection was performed, with a safety margin, in addition to chemotherapy and radiotherapy, with no signs of recurrence after 4 year of follow-up.

## Discussion

Pleomorphic adenoma is the most common salivary glands tumor, whether in major or minor salivary glands (5). CExPA is a rare, aggressive, poorly understood malignancy that occurs in the salivary glands, arising from a primary or recurrent benign pleomorphic adenoma (6). The diagnosis of CExPA is primarily based on the unequivocal identification of malignant carcinomatous components in a typical pleomorphic adenoma (2). However, the amount of carcinomatous component in CExPA is variable, comprising between less than 30% to more than 84%, although in certain cases it can even represent the entire neoplasm, so that the identification of the PA is only possible based on previous biopsies or clinicopathologic correlation (3). In the current case, the sudden change in tumor biological behavior after two years evolution was suggestive of malignant transformation in a pre-existing benign tumor (3). Furthermore, significant smoking history, rapid growth and tumors larger than 2 cm, as also observed in the current case, are considered risk factors for malignant transformation of pleomorphic adenoma (7).

Despite the relevance of the clinical history, pathological analysis is necessary to assure the malignant transformation of a pre-existing pleomorphic adenoma. As the clinical course of CExPA differ according to cellular differentiation, reporting the histological subtype is important to assess potential biomarkers in diagnostic and therapeutic trials (2). In the current case, the histological features usually seen in a typical PA component was unequivocal, and composed of abundant myoepithelial cells and duct-like structures with double cell layer lining, as well as stromal connective tissue with hyaline and myxoid changes (8). On the other hand, the mucous, intermediate and atypical squamous differentiation indicated the arising of mucoepidermoid carcinoma (MEC) (9). The presence of sialomucin content of mucous-producing cells was confirmed by mucicarmine and Alcian blue staining (10). Due to the paucity of cystic/microcystic formation (<25%) and pattern of tumor infiltration in small nests and islands observed in the current case, the MEC was classified as high grade according to Brandwein *et al*. (11). The tumor immunohistochemical profile, expressed by positive immunostaining for CK7 and CK14 is used to identify primary salivary gland neoplasms, whereas CK13 and p63 staining, taken together, are helpful to differentiate MEC from other salivary gland tumors (12,13). We found that ki-67 immunoexpression in PA areas was lower than 1% whereas MEC showed proliferation index of about 30%. These data suggest the apparent existence of two different tumor components, with different proliferative potential, supporting the thesis of malignant transformation in a pre-existing benign tumor (14).

Treatment of CExPA often involves a surgical procedure and post-operative adjuvant radiation therapy, but the 5-year survival rate, ranging from approximately 25% to 65%, is closely related to metastatic disease (15). Likewise, the tumor was subjected to complete excision and further controlled by post-operative radiation in the current case. Furthermore, the clinical TNM-classification was T4N0Mx, indicating an early diagnosis of the malign transformation, and suggesting a better prognosis. Although no recurrence was observed so far, a longer-term follow-up is required to assure that tolerable local tumor control was effectively achieved.

In conclusion, CExPA is a rare aggressive malignancy whose diagnosis can be challenging not only because the PA areas of the tumor is commonly abundant and overestimated, whereas the malignant component may be difficult to be identified, but also due to the frequent overlap in histopathological features with other salivary gland tumors. The diagnosis can be achieved by properly correlating the proper correlation between clinical, pathological and immunohistochemical features. Even considering the rarity of this malignant tumor, clinicians should be aware of changes in biological behavior of apparent benign tumors to assure an early diagnosis and a better prognosis.
